# Chemical Profile, Antioxidative, and Gut Microbiota Modulatory Properties of Ganpu Tea: A Derivative of Pu-erh Tea

**DOI:** 10.3390/nu12010224

**Published:** 2020-01-15

**Authors:** Yuying Zheng, Xuan Zeng, Tingting Chen, Wei Peng, Weiwei Su

**Affiliations:** Guangdong Engineering & Technology Research Center for Quality and Efficacy Reevaluation of Post-Market Traditional Chinese Medicine, Guangdong Provincial Key Laboratory of Plant Resources, School of Life Sciences, Sun Yat-sen University, Guangzhou 510275, China; vicky_0224@126.com (Y.Z.); zengx6@mail2.sysu.edu.cn (X.Z.);

**Keywords:** Ganpu tea, chemical profile, antioxidant activity, gut microbiota modulatory

## Abstract

Ganpu tea is an emerging tea drink produced from Pu-erh tea and the pericarp of *Citrus reticulate* Chachi (GCP). Recently, it has been increasingly favored by consumers due to the potential health effects and special taste. However, information concerning its chemical profile and biological activities is scarce. In this work, a total of 92 constituents were identified in hot-water extracts of Ganpu tea with ultra-high performance liquid chromatography/quadrupole-time-of-flight tandem mass spectrometry (UHPLC-Q-TOF-MS/MS). Moreover, the antioxidative and gut microbiota modulatory properties of Ganpu tea were investigated in rats after long-term dietary consumption. Ganpu tea and GCP could significantly enhance the activities of superoxide dismutase (SOD) by 13.4% (*p* < 0.05) and 15.1% (*p* < 0.01), as well as the activities of glutathione peroxidase (GSH-Px) by 16.3% (*p* < 0.01) and 20.5% (*p* < 0.01), respectively. Both showed better antioxidant capacities than Pu-erh tea. Ganpu tea increased the abundance of *Bifidobacterium*, *Lactobacillus*, and *Lactococcus*, suggesting the potential of Ganpu tea in modulating the gut microbiota to benefit human health. The obtained results provide essential information for further investigation of Ganpu tea.

## 1. Introduction

**Figure 1:**
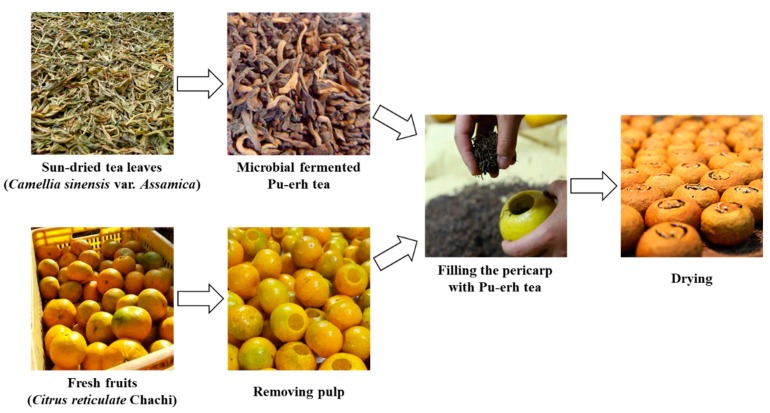
Manufacturing process of Ganpu tea.

Pu-erh tea is a microbial fermented tea produced using sun-dried leaves of large-leaf tea species (*Camellia sinensis* (Linn.) var. *assamica* (Masters) Kitamura) in Yunnan Province of China [1]. It has been proven to possess multiple health-promoting effects, including antioxidation, anti-aging, and hypolipidemic efficacies [2,3]. As a well-known tea, Pu-erh tea is increasingly popular among consumers in Southeast Asia and has many derivative products. Ganpu tea is an emerging tea drink produced from Pu-erh tea and the pericarp of *Citrus reticulate* Chachi from Xinhui County (Guangdong Province, China) [4]. The manufacturing process of Ganpu tea is briefly summarized as follows: Whole fresh pericarp of *C. reticulate* Chachi is separated, filled with Pu-erh tea, dried together, and stored in a cool and ventilated place (Figure 1). The pericarp of *C. reticulate* “Chachi” from Xinhui County, called “Guangchenpi” (GCP) in Chinese, is widely used in cuisine and traditional medicine in China mainly due to its beneficial health effects as well as its special flavor [5,6]. Traditionally, both GCP and Pu-erh tea are considered to be of better quality if they are stored longer [1,7]. For instance, only GCP that has been stored for more than three years can be considered qualified [8]. The combination of Ganpu tea not only incorporates this characteristic but also blends the fruit flavor of GCP with the mellow taste of Pu-erh tea. Thanks to its health efficacies and special taste, Ganpu tea is increasingly favored by consumers and therefore its market demand has expanded rapidly in China. However, investigations concerning the chemical profile and biological activities of Ganpu tea are scarce.

Gut microbiota play a vital role in many aspects of human nutrition and health [9], including promoting the supply of nutrients, preventing pathogen colonization, and shaping and maintaining normal mucosal immunity [10]. Gut microbiota dysbiosis (generally with lower bacterial diversity) has been reproducibly observed in animal models and human studies with multiple diseases, such as obesity [11], diabetes [12], chronic gastrointestinal disease [13], etc. Certain foods and dietary patterns can influence the diversity and abundance of gut microbiota, which consequentially in turn affect host health. Recently, gut microbiota has emerged as a new frontier in understanding the health efficacies of functional foods and complementary medicines [14]. Both Pu-erh tea and GCP contain abundant polyphenolic compounds, which may be associated with their beneficial health properties [1,5]. After oral administration, these polyphenolic compounds will unavoidably interact with gut microbiota by modification of the microbial composition or by conversion of the phenolics to further bioactive compounds [15]. Therefore, it would be interesting to investigate whether and how Ganpu tea rich in phenolic compounds may alter gut microbiota.

This study was conducted to investigate the chemical profiles and antioxidative properties of Ganpu tea, along with its modulatory effects on gut microbiota. The chemical composition in the water extract of Ganpu tea was first profiled using a UHPLC-Q-TOF-MS/MS system. Then, oral administration experiments with the water extract of Ganpu tea were conducted in rats. The activities of antioxidant enzymes in serum were determined to evaluate the antioxidative properties of Ganpu tea. Moreover, the gut microbial community was analyzed by high throughput 16S rRNA gene sequencing.

## 2. Materials and Methods

### 2.1. Herbal Material and Chemicals

Samples of Ganpu tea and the corresponding raw materials (including Pu-erh tea and GCP) were provided by Xinhui Hele Tea Art Co. Ltd. (Jiangmen, China). The reference standards of gallic acid, caffeine, hesperidin, naringin, neohesperidin, rutin, rhoifolin, and synephrine were obtained from the National Institute for Control of Biological and Pharmaceutical Products of China (Beijing, China). Naringenin, poncirin, nobiletin, sinensetin, tangeretin, and mass spectrometry (MS) grade formic acid were purchased from Sigma-Aldrich (St. Louis, MO, USA). Hesperetin and *N*-methyltyramine were acquired from Sinova (Shenzhen, China). MS-grade acetonitrile was purchased from Fisher Scientific (Pittsburgh, PA, USA). Water used in the experiment was distilled and further purified with a Milli-Q system (Millipore, Milford, MA, USA). All other reagents used were of analytical grade.

### 2.2. Sample Preparation

The Ganpu tea sample (100 g) was cut into small pieces, and then soaked in boiled distilled water for three times (2, 1.5, and 1.5 L of each bulk, respectively, for 20, 15, and 15 min, respectively). After filtration, the whole extracts were evaporated to 500 mL by a rotary evaporator (Eyela, Tokyo, Japan) at 60 °C to obtain the Ganpu tea extract (GTE) with a concentration of 0.2 g/mL. As the whole Ganpu tea (rather than its crumb) is usually put into hot water when brewing, the average weight ratio of Pu-erh tea to GCP in Ganpu tea was experimentally determined as 8:2. To separately evaluate the effects of Pu-erh tea and GCP, a corresponding proportion of raw materials were extracted with boiled distilled water and concentrated to obtain Pu-erh tea extract (PTE) (0.16 g/mL) and GCP extract (GCPE) (0.04 g/mL). The final extracts were stored at −80 °C until further utilization.

The mixed solution of reference standards used in identification was prepared with methanol at the concentration of 10 μg /mL for each compound. The extracted solutions were filtered through a 0.22-μm microporous filter before UHPLC-Q-TOF-MS/MS analysis.

### 2.3. UHPLC-Q-TOF-MS/MS Analysis

Analysis of the Ganpu tea sample was performed using UHPLC-Q-TOF-MS/MS, ultra-fast liquid chromatography (Shimadzu Corp., Kyoto, Japan) coupled with quadrupole/time-of-flight mass spectrometry (Triple TOF 5600 plus, AB SCIEX, Foster City, CA, USA). Gradient chromatographic separation was performed on a Kinetex C_18_ column (2.6 μM, 150 mm × 3.0 mm) and maintained at 40 °C. The mobile phase consisted of acetonitrile (A) and water containing 0.1% aqueous formic acid (*v*/*v*) (B). The elution was carried out by the following program: 10–30% A (0–5 min), 30–80% A (5–27 min), 80–100% A (27–28 min), and 100% A (28–33 min) with the flow rate kept at 0.3 mL/min. The injection volume was 10 μL.

MS/MS identification was conducted using an electrospray ionization (ESI) source with the following parameters. The ion spray voltage was 5500 V in positive ion mode while −4500 V in negative ion mode. The mass range was from *m*/*z* 100 to 1500. The ion source gas 1 and gas 2 were both 55 psi, and the curtain gas was set as 35 psi. The ion source temperature was maintained at 550 °C. The declustering potential was 80 V. The collision energy and its spread was set as 35 and 25 eV, respectively. Nitrogen was used as the nebulizer and auxiliary gas. Data acquisition was carried out using Analyst^®^ TF 1.6 software (AB Sciex, Foster City, CA, USA) in the information-dependent acquisition mode.

### 2.4. Animals and Experimental Design

Male Sprague-Dawley rats (weighing 180–220 g) were purchased from Guangdong Medical Experimental Animal Center and raised in the specific pathogen-free (SPF) condition. All experimental processes were approved by the Animal Ethics Committee of the School of Life Sciences in Sun Yat-sen University, and conducted according to the National Institutes of Health guide for the care and use of laboratory animals (NIH Publications No. 8023, revised 1978). Environmental conditions in the SPF houses was kept at 20 to 23 °C, 50% to 65% relative humidity, and 12-h dark/light cycle. Rats were fed for one week to adapt to the new environment before the experiments.

In total, 40 rats were randomly assigned into four groups with 10 rats in each group: Control group, GTE group (0.2 g/mL, 15 mL/kg/d), PTE group (0.16 g/mL, 15 mL/kg/d), and GCPE group (0.04 g/mL, 15 mL/kg/d). Rats were administrated a corresponding extract by gavage twice daily for 28 consecutive days. The control group received the same volume of distilled water. After the last administration, the animals were fasted for 12 h with water available ad libitum. On the next day, the rats were anesthetized with 10% chloral hydrate (3 mL/kg) by intraperitoneal injection. Blood samples were collected from the abdominal artery and then centrifuged to obtain serum samples. Feces samples were collected from the rectum of each rat, transferred into sterile conical tubes, and immediately frozen in liquid nitrogen. Obtained samples were stored at −80 °C until further analysis.

### 2.5. Assay of Antioxidant Enzyme Activity in Serum

Activities of serum antioxidant enzymes, including superoxide dismutase (SOD), malondialdehyde (MDA), and glutathione peroxidase (GSH-Px), were determined in accordance with the protocols of the corresponding kits (Nanjing Jiancheng Bioengineering Institute, Nanjing, China) with a spectrophotometer.

### 2.6. Gut Microbiota Analysis Using 16S rRNA Gene Sequencing

Total bacterial DNA were extracted from feces samples using the Power Soil DNA Isolation Kit (MO BIO Laboratories, USA) according to the manufacturer’s instructions. The 16S rRNA gene comprising V3–V4 regions was amplified by PCR using the common primers 338F (5′-ACTCCTACGGGAGGCAGCA-3′) and 806R (5′-GGACTACHVGGGTWTCTAAT-3′) combined with adapter sequences and barcode sequences. After PCR amplification, sequencing was performed on an Illumina Hiseq 2500 platform by Biomarker Technologies Co. Ltd. (Beijing, China).

The raw paired-end reads were merged using FLASH (version 1.2) [16] and filtered with Trimmomatic (version 1.2.11) [17]. All quality filtered sequencing reads were then clustered into operational taxonomic units (OTUs) based on a 97% sequence similarity according to UCLUST [18]. The OTU abundance information was normalized for further analyses of the alpha and beta diversity. Community richness and diversity estimators of Chao1, ACE, Shannon index, and Simpson index were calculated in Quantitative Insights into Microbial Ecology (QIIME) program (version 1.8) [19]. Furthermore, beta diversity analysis was utilized to evaluate the differences of samples in species complexity, which was proceeded by the gower algorithm in principal coordinate analysis (PCoA). To identify the representative taxa among each group, the linear discriminant analysis (LDA) effect size (LEfSe) algorithm was then performed with an alpha value of 0.05 and an LDA score threshold of 3.0 [20]. All processes were performed on the BMKCloud platform (www.biocloud.net).

### 2.7. Statistical Analysis

Data were expressed as mean ± standard deviation (SD). The significant differences between the groups were assessed by Student’s *t*-test in SPSS 18.0, and *p* < 0.05 was considered as a significant difference.

## 3. Results and Discussion

### 3.1. Identification of Chemical Compounds in GTE by UHPLC-Q-TOF-MS/MS

**Figure 2:**
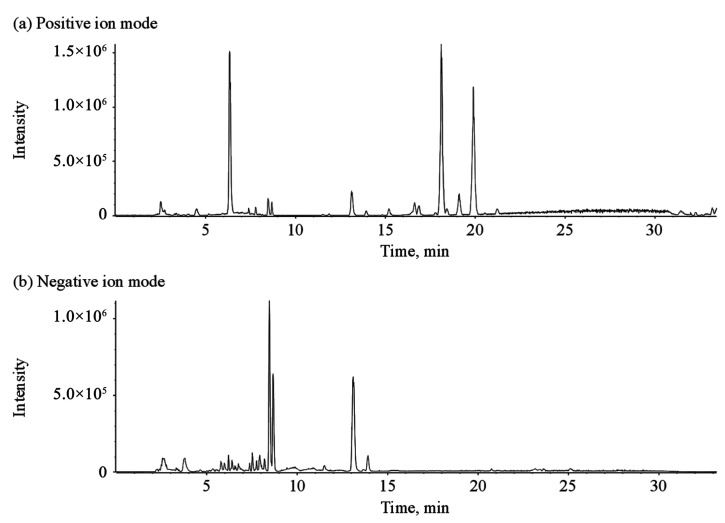
The basic peak chromatograms of Ganpu tea extract (GTE) in positive (**a**) and negative (**b**) ion modes.

The chemical compounds of GTE, as well as PTE and GCPE, were characterized using UHPLC-Q-TOF-MS/MS in both positive and negative ion modes. The basic peak chromatograms (BPCs) of GTE are shown in Figure 2. The elution time, accurate molecular weights, and MS/MS fragment ions of the identified compounds are presented in Table 1. A total of 92 compounds were identified or tentatively characterized, including 63 flavonoids, 8 catechins, 14 organic acids, 6 alkaloids, and 1 limonin.

Flavonoids are an important class of plant secondary metabolites and have been shown to possess multiple biological activities, including antioxidant, anti-inflammatory, and cardioprotective properties [21,22]. In this work, a total of 63 flavonoids—comprising 12 flavonoid-*O*-glycosides, 7 flavonoid-*C*-glycosides, 11 flavonoid aglycones, and 33 polymethoxylated flavonoids—were detected in GTE. These flavonoids were mainly derived from GCP, and the corresponding MS/MS fragmentation modes were aligned with our reported results [6].

**Figure 3:**
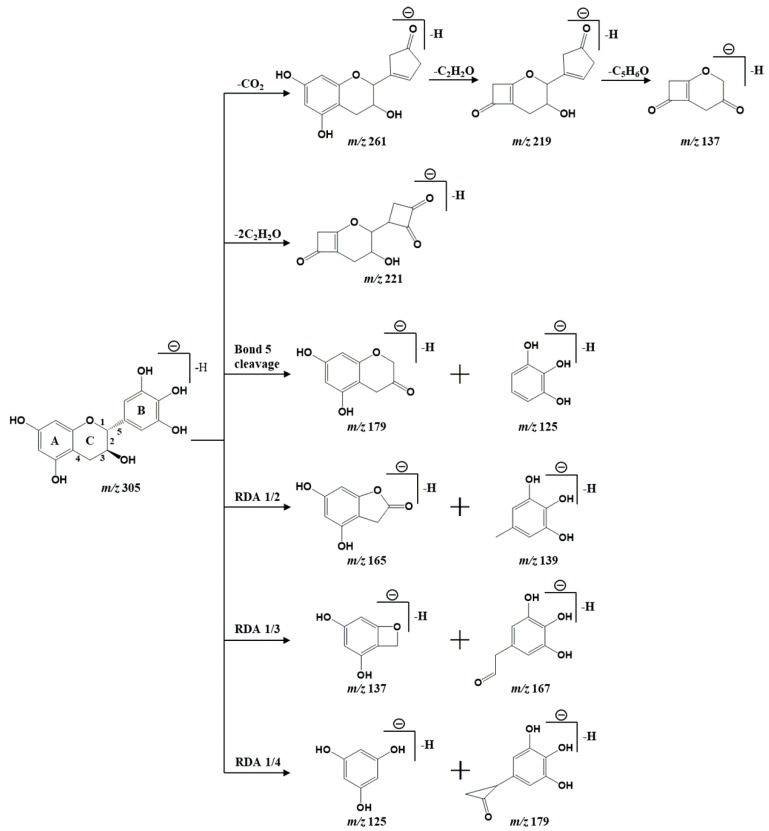
Proposed fragmentation scheme of deprotonated gallocatechin.

Catechins, a class of polyphenolic compounds present in tea, are regarded as a major contributor to the beneficial effects of tea diets. A total of eight catechins (compounds **6**, **12**, **17**, **22**, **23**, **28**, **33**, and **35**) were characterized in GTE. Compound 6 and 12 both gave the quasi-molecular ions [M-H]^−^ at *m*/*z* 305 and have similar fragmentation patterns. By comparing the accurate molecular weights, MS/MS fragmentation modes, and elution time with the references [23], compound 6 and 12 were proposed as gallocatechin and epigallocatechin, respectively. Typical retro-Diels–Alder (RDA) reactions were observed in the MS/MS fragmentation of these catechins. For example, with the RDA reaction involved in the cleavage of bonds 1 and 2 in the C ring, deprotonated gallocatechin (*m*/*z* 305.0664) gave its product ions at *m*/*z* 165.0119 and 139.0398 while *m*/*z* 167.0326 and 137.0228 were considered to flow from the cleavage of bonds 1 and 3. The signals at *m*/*z* 179.0447 and 125.0236 could be explained by either the cleavage of bond 5 or the simultaneous cleavage of bonds 1 and 4. In addition, the product ions at *m*/*z* 261.0807, 219.0621, and 137.0228 were yielded by the successive loss of CO_2_, C_2_H_2_O, and C_5_H_6_O from deprotonated gallocatechin. Another product ion at *m*/*z* 221.0413 was generated from the successive loss of C_2_H_2_O in ring A and B. The fragmentation scheme of deprotonated gallocatechin is proposed in Figure 3.

### 3.2. Antioxidant Activities

**Figure 4:**
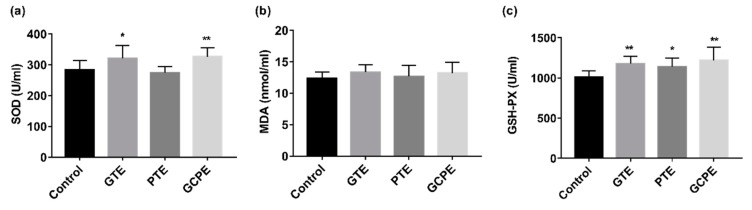
Effects of oral administration of GTE, PTE, and GCPE on the antioxidant functions in rats (*n* = 10, compare with the control group * *p* < 0.05, ** *p* < 0.01). (**a**) Superoxide dismutase (SOD) in serum; (**b**) malondialdehyde (MDA) content in serum; (**c**) glutathione peroxidase (GSH-PX) in serum.

Oxidative stress is important in the pathogenesis of many diseases, such as diabetes, cardiovascular diseases, and neurodegenerative diseases [24]. Plant-derived polyphenolic compounds are an important class of dietary antioxidant components that may reduce the risk of these diseases [25]. Flavonoids and catechins are primary polyphenols found in GTE, which are mainly derived from GCP [26] and Pu-erh tea [1], respectively. As shown in Figure 4, both the GTE group and GCPE group could significantly increase the activities of antioxidant enzymes SOD and GSH-PX. Specifically speaking, Ganpu tea significantly enhanced the activities of SOD and GSH-PX by 13.4% (*p* < 0.05) and 16.3% (*p* < 0.01) while GCP increased their activities by 15.1% (*p* < 0.01) and 20.5% (*p* < 0.01), respectively. Correspondingly, the PTE group only significantly increased the activity of GSH-PX by 12.4% (*p* < 0.05). There were no significant differences in the content of MDA among the four groups of rats. The results showed that GTE and GCPE had better antioxidant capacities than PTE, suggesting that Ganpu tea and GCP may serve as natural dietary antioxidants (especially containing flavonoids).

### 3.3. Effects on Fecal Bacteria Composition

After 28 days of dietary intervention, feces samples were collected for an analysis of the gut microbiota community in the rats. A total of 2,542,728 high-quality sequences of the V3–V4 region of the 16S rRNA were obtained from 40 feces samples. The average sequence number was 66,914. Based on a 97% similarity level, all of the effective reads were clustered into OTUs. At the phylum level, the most abundant bacteria were *Firmicutes* (42.4%), *Bacteroidetes* (40.0%), *Proteobacteria* (12.3%), *Actinobacteria* (2.5%), and *Cyanobacteria* (0.95%).

**Figure 5:**
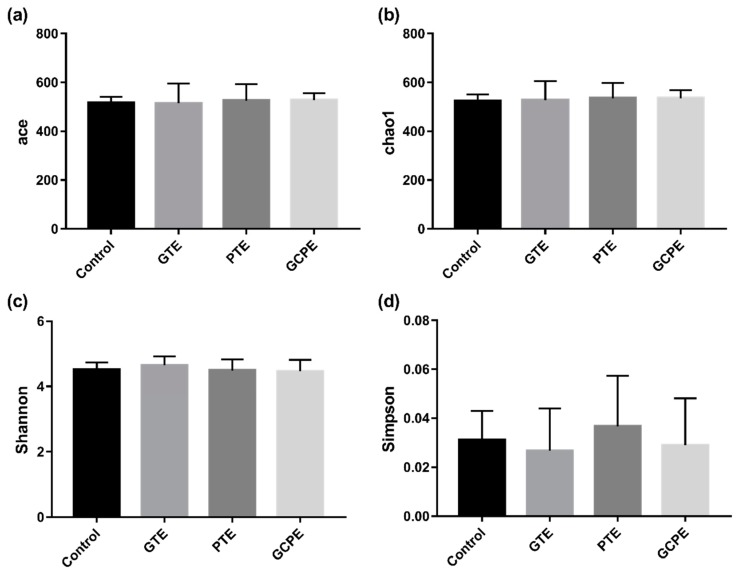
The diversity index, including ACE (**a**), Chao1 (**b**), Shannon (**c**) and Simpson index (**d**), of gut microbiota in rats in different groups (*n* = 10).

**Figure 6:**
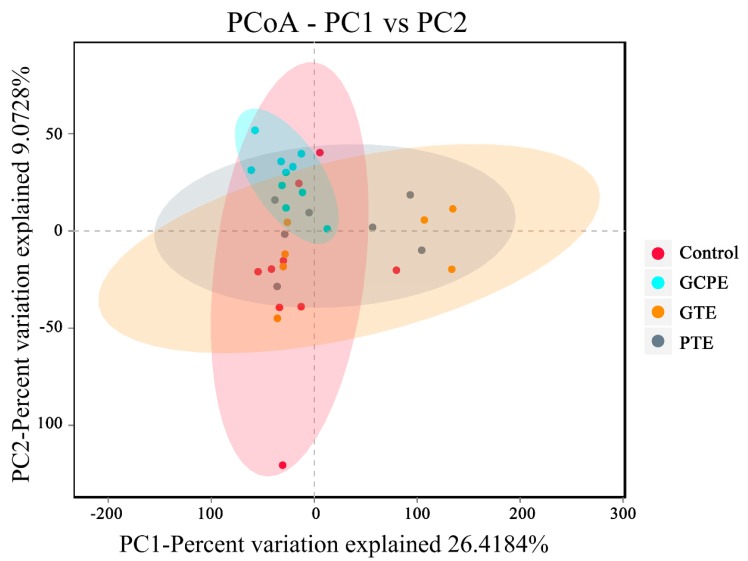
PCoA plots of microbial communities were based on operational taxonomic units (OTUs) composition, each treatment group is represented by a different color (*n* = 10).

**Figure 7:**
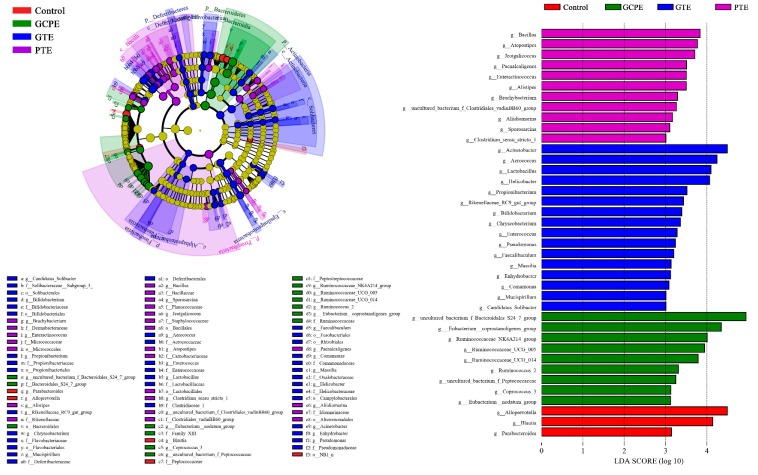
Differences in the gut microbiota between groups using linear discriminant analysis effect size (LEfSe) analysis at the genus level (*n* = 10). For taxa, which were defined as unclassified, no rank, ncultured, or Incertae-Sedis, the name of a higher taxon level was added before its taxon abbreviation (*p*, phylum; c, class; o, order; f, family; g, genus; s, species).

After oral administration for 28 consecutive days, there was no significant differences in bacterial richness (expressed by the ACE and Chao1 index in Figure 5), diversity (expressed by the Shannon and Simpson index in Figure 5), and overall structure (shown in Figure 6) among the four groups. These results indicated that long-term consumption of GTE, PTE, or GCPE did not significantly affect the gut microbiota in healthy rats. Nevertheless, some subtle beneficial changes in gut microbiota, which were associated with the dietary consumption, were identified with the LEfSe analysis. As the threshold on the logarithmic LDA score was set at 3, a total of 39 genera were screened in the LEfSe analysis (shown in Figure 7).

Pu-erh tea is processed by microbial fermentation of sun-dried green tea leaves. Microbes play important roles in the development of the special properties of fermented Pu-erh tea [1]. Some bacteria that are common in the fermentation process of Pu-erh tea were also found to be enriched in the gut microbiota of rats in the PTE group, including *Bacillus*, *Brachybacterium*, *Paenalcaligenes*, and *Jeotgalicoccus*. Among them, *Bacillus* is a safe thermoduric probiotic and the dominant bacterium during the fermentation of Pu-erh tea [27]. *Bacillus* could produce β-glucosidase and cellulase, which would decompose the nutrients of the tea, produce organic acids, and further promote the formation of flavor substances in Pu-erh tea. *Bacillus* can facilitate the digestion and utilization of nutrients in the host as well. Similarly, *Brachybacterium* is also a cellulose-decomposing bacterium [28]. These bacteria possess essential capacities for the fermentation of non-digestible carbohydrates (e.g., dietary fibers), which are defined as food components or ingredients that are not digestible by the host [9]. This fermentation gives rise to a mass of sugar or amino acid components, which could specifically or selectively nourish beneficial colonic micro-organisms like *Bifidobacterium* [29].

Moreover, Pu-erh tea stimulated the relative abundance of *Alistipes* and *Odoribacter* in the gut microbiota of rats. In several studies, a significant increase of *Alistipes* and *Odoribacter* was found to be associated with a polyphenol-rich diet [30,31]. The major metabolic product of *Alistipes* is succinic acid [23] while *Odoribacter* is a butyric acid-producing bacterium [32]. These short-chain fatty acids (SCFAs), which are metabolic by-products of bacterial metabolism, play a vital role in the maintenance of colonic integrity and metabolism [33]. The available literature indicates that SCFAs could act not only as nutrients for the colonic epithelium, and modulators of intracellular pH and cell volume, but also as regulators of proliferation, differentiation, and gene expression [34].

In the GCPE group, nine genera were upregulated, with four of them belonging to the Ruminococcaceae family. Ruminococcaceae members possess a capacity to degrade plant fibers and polysaccharides to generate glucose and SCFAs, which may promote the energy intake from fiber, inhibit opportunistic pathogens, and protect the hosts against inflammation and colonic diseases [35,36]. Some studies indicated that the abundance of Ruminococcaceae family bacteria correlates negatively with arterial stiffness and endotoxemia [37,38]. Besides, the *Coprococcus 3* and *Eubacterium coprostanoligenes* group were enriched within the GCPE group. *Coprococcus 3* is a butyrate-producing genus, which could stimulate colonic motility to maintain a healthy gut [39]. The *Eubacterium coprostanoligenes* group can convert cholesterol to coprostanol, which is poorly absorbed in human intestines and would be excreted, leading to a reduction in the blood cholesterol concentration [40].

With the LEfSe analysis, 16 genera were found to be enriched within the GTE group. In particular, *Bifidobacterium*, *Lactobacillus*, and *Lactococcus*, which are well-known probiotics, were abundant in the gut microbiota of rats within the GTE group. *Lactobacillus* and *Bifidobacterium* are engaged in many functional foods and dietary supplements, with many health-promoting properties, such as the prevention of enteropathogen colonization (barrier effects) [41], reinforcement of the immune system [42,43,44], and easing digestive concerns [45]. *Lactococcus lactis* is a food-grade microorganism as well, and has been widely used in the food fermentation industry. Research showed that *Lactococcus lactis* exhibits anti-inflammatory properties in colitis [46]. *Faecalibaculum* was upregulated in the GTE group as well, which could produce butyrate [47]. Meanwhile, some pathogens, like *Acinetobacter* and *Pseudomonas*, were detected in the GTE group, which have been discovered in the fermentation of Pu-erh tea [27,48]. Whether they are harmless symbionts or pathogens needs further study.

Taken together, long-term dietary intake of GTE, PTE, or GCPE exerts some subtle beneficial effects in the gut microbiota of healthy rats. In particular, Ganpu tea, the mixture of Pu-erh tea and GCP, had a better capacity to stimulate the proliferation of probiotics, including *Bifidobacterium*, *Lactobacillus*, and *Lactococcus*. These results reveal the potential of Ganpu tea in modulating host gut microbiota, and consequentially, improving health benefits.

## 4. Conclusions

In this study, the chemical profiles of Ganpu tea were first investigated with a rapid UHPLC-Q-TOF-MS/MS method. A total of 92 compounds were identified or tentatively characterized, including 63 flavonoids, 8 catechins, 14 organic acids, 6 alkaloids, and 1 limonin. Furthermore, the antioxidative and gut microbiota modulatory properties of Ganpu tea were evaluated in rats after oral administration for 28 consecutive days. Ganpu tea and GCP were found to possess better antioxidant capacities than Pu-erh tea. Moreover, Ganpu tea could enhance the abundance of probiotics (including *Bifidobacterium*, *Lactobacillus*, and *Lactococcus*), revealing the potential of Ganpu tea in modulating the host gut microbiota to benefit human health. The obtained results would be useful in improving the utilization and consumption of Ganpu tea in functional foods/beverages, and provide essential information for further research to investigate the underlying biochemical mechanisms of its health efficiencies.

## Figures and Tables

**Table 1:** Identification of the chemical compounds of Ganpu tea by ultra-high performance liquid chromatography/quadrupole-time-of-flight tandem mass spectrometry (UHPLC-Q-TOF-MS/MS).

No.	Identification	Molecular Formula	Retention Time (min)	[M+H]^+^ (Error, ppm)	[M-H]^−^ (Error, ppm)	Fragment Ions in the Positive Ion Mode (*m*/*z*) ^b^	Fragment Ions in the Negative Ion Mode (*m*/*z*) ^b^	Source
Organic Acids and Their Esters								
1	Quinic acid	C_7_H_12_O_6_	2.60	ND ^d^	191.0205 (1.5)	ND	173.0446 [M-H-H_2_O]^−^, 127.0388, 85.0309	Puerh tea
3	3-Glucogallic acid/4-Glucogallic acid	C_13_H_16_O_10_	3.14	ND	331.0673 (0.7)	ND	169.0114 [M-H-Glc]^−^, 125.0219 [M-H-Glc-CO_2_]^−^	Puerh tea
4	Theogallin	C_14_H_16_O_10_	3.51	345.0817 (−0.3)	343.0673 (0.8)	153.0184 [M+H-C_7_H_12_O_6_]^+^	191.0545 [M-H-C_7_H_4_O_4_]^−^, 169.0113 [M-H-C_7_H_10_O_5_]^−^	Puerh tea
5	Gallic acid ^a^	C_7_H_6_O_5_	3.76	171.0286 (−1.1)	169.0152 (3.4)	153.0175 [M+H-H_2_O]^+^, 135.0063 [M+H-2H_2_O]^+^, 125.0230 [M+H-H_2_O-CO]^+^, 107.0135 [M+H-2H_2_O-CO]^+^, 81.0356	125.0239 [M-H-CO_2_]^−^	Puerh tea
7	3-Caffeoylquinic acid	C_16_H_18_O_9_	5.00	355.1022 (3.6)	353.0876 (0.8)	163.0385 [M+H-C_7_H_12_O_6_]^+^, 135.0430 [M+H-C_7_H_12_O_6_-CO]^+^,	191.0549 [M-H-C_9_H_7_O_3_]^−^, 179.0338 [M-H-C_7_H_10_O_5_]^−^, 135.0441 [M-H-C_7_H_10_O_5_-CO_2_]^−^	Puerh tea
9	Vanillic acid ^c^	C_8_H_8_O_4_	5.29	ND	167.0357 (3.7)	ND	152.0104 [M-H-CH_3_]^−^, 108.0242 [M-H-C_2_H_5_]^−^	GCP
14	Caffeoyl-glucose	C_15_H_18_O_9_	5.75	ND	341.0881 (0.9)	ND	179.0339 [M-H-Glc]^−^, 161.0568 [M-H-Glc-H_2_O]^−^, 135.0435 [M-H-Glc-CO_2_]^−^	GCP, Puerh tea
15	3-*p*-Coumaroylquinic acid	C_16_H_18_O_8_	5.83	339.1074 (−1)	337.0932 (1)	147.0437 [M+H-C_7_H_10_O_5_-H_2_O]^+^, 119.0495 [M+H-C_9_H_8_O_2_-CO_2_-CO]^+^, 91.0564 [M+H-C_7_H_10_O_5_-HCOOH-CO]^+^	191.0544 [M-H-C_9_H_6_O_2_]^−^, 163.0384 [M-H-C_7_H_10_O_5_]^−^, 119.0511 [M-H-C_7_H_10_O_5_-CO_2_]^−^,	Puerh tea
16	4-Caffeoylquinic acid	C_16_H_18_O_9_	5.94	355.1021 (0)	353.0874 (0.8)	163.0387 [M+H-C_7_H_12_O_6_]^+^, 145.0277, 135.0425 [M+H-C_7_H_12_O_6_-CO]^+^,	191.0547 [M-H-C_9_H_6_O_3_]^−^, 179.0348 [M-H-C_7_H_10_O_5_]^−^, 173.0442 [M-H-C_9_H_8_O_4_]^−^, 135.0456 [M-H- C_7_H_10_O_5_-CO_2_]^−^, 93.0364	Puerh tea
24	5-*p*-Coumaroylquinic acid	C_16_H_18_O_8_	6.80	339.1071 (−0.6)	337.0937 (2.5)	147.0444 [M+H-C_7_H_10_O_5_-H_2_O]^+^, 119.0500 [M+H-C_9_H_8_O_2_-CO_2_-CO]^+^, 91.0555 [M+H-C_7_H_10_O_5_-HCOOH-CO]^+^	173.0444 [M-H-C_9_H_6_O_2_-H_2_O]^−^ 163.0384 [M-H-C_7_H_10_O_5_]^−^, 119.0498 [M-H-C_7_H_10_O_5_-CO_2_]^−^,	Puerh tea
25	Caffeic acid ^c^	C_9_H_8_O_4_	6.85	ND	179.0362 (4.5)	ND	135.0443 [M-H-CO_2_]^−^	GCP, Puerh tea
26	4-*p*-Coumaroylquinic acid	C_16_H_18_O_8_	6.93	339.1074 (0.1)	337.0936 (2.5)	147.0447 [M+H-C_7_H_10_O_5_-H_2_O]^+^, 119.0497 [M+H-C_9_H_8_O_2_-CO_2_-CO]^+^, 91.0558 [M+H-C_7_H_10_O_5_-HCOOH-CO]^+^	191.0548 [M-H-C_9_H_6_O_2_]^−^, 173.0446 [M-H-C_9_H_6_O_2_-H_2_O]^−^ 163.0366 [M-H-C_7_H_10_O_5_]^−^, 119.0504 [M-H-C_7_H_10_O_5_-CO_2_]^−^,	Puerh tea
38	*p*-Coumaric acid	C_9_H_8_O_3_	8.23	ND	163.0411 (2.2)	ND	119.0503 [M-H-CO_2_]^−^	Puerh tea
46	Ferulic acid ^a,c^	C_10_H_10_O_4_	8.69	ND	193.0508 (0.7)	ND	178.0273 [M-H-CH_3_]^−^, 134.0360 [M-H-CH_3_-CO_2_]^−^,	GCP
**Flavan-3-ols**								
6	Gallocatechin	C_15_H_14_O_7_	4.59	307.081 (−0.6)	305.0664 (2.1)	195.0637 [M+H-CO-2C_2_H_2_O]^+^, 177.0532 [M+H-CO-2C_2_H_2_O-H_2_O]^+^, 139.0388 [M+H-C_8_H_8_O_4_]^+^	261.0807 [M-H-CO_2_]^−^, 221.0413 [M-H-2C_2_H_2_O]^−^, 219.0621 [M-H-CO_2_-CH_2_O]^−^, 179.0447 [M-H-C_6_H_6_O_3_]^−^, 167.0326 [M-H-C_7_H_6_O_3_]^−^, 165.0199 [M-H-C_7_H_8_O_3_]^−^, 139.0398 [M-H-C_8_H_6_O_4_]^−^, 137.0228 [M-H- CO_2_-CH_2_O-C_5_H_6_O]^−^, 125.0236 [M-H-C_9_H_8_O_4_]^−^	Puerh tea
12	Epigallocatechin	C_15_H_14_O_7_	5.37	307.0815 (0.9)	305.0678 (1.5)	195.1763 [M+H-CO-2C_2_H_2_O]^+^, 177.0544 [M+H-CO-2C_2_H_2_O-H_2_O]^+^, 139.0387 [M+H-C_8_H_8_O_4_]^+^	261.0784 [M-H-CO_2_]^−^, 221.0439 [M-H-2C_2_H_2_O]^−^, 219.0667 [M-H-CO_2_-CH_2_O]^−^, 179.0350 [M-H-C_6_H_6_O_3_]^−^, 167.0242 [M-H-C_7_H_6_O_3_]^−^, 165.0178 [M-H-C_7_H_8_O_3_]^−^, 137.0234 [M-H- CO_2_-CH_2_O-C_5_H_6_O]^−^, 125.0245 [M-H-C_9_H_8_O_4_]^−^	Puerh tea
17	Catechin	C_15_H_14_O_6_	5.99	291.0865 (0.6)	289.0727 (2.5)	207.0652 [M+H-C_4_H_4_O_2_]^+^, 179.0679 [M+H-C_4_H_4_O_2_-CO]^+^ 139.0396 [M+H-C_8_H_8_O_3_]^+^, 123.0451 [M+H-C_8_H_8_O_4_]^+^	245.0818 [M-H-CO_2_]^−^, 205.0504 [M-H-2C_2_H_2_O]^−^, 203.0703 [M-H-CO_2_-C_2_H_2_O]^−^, 137.0244 [M-H-C_8_H_8_O_3_]^−^	Puerh tea
22	Epicatechin	C_15_H_14_O_6_	6.61	291.0866 (0.9)	289.0727 (3.4)	207.0649 [M+H-C_4_H_4_O_2_]^+^, 147.0441 [M+H-C_4_H_4_O_2_-C_2_H_2_O-H_2_O]^+^, 139.0397 [M+H-C_8_H_8_O_3_]^+^, 123.0452 [M+H-C_8_H_8_O_4_]^+^	245.0839 [M-H-CO_2_]^−^, 205.0505 [M-H-2C_2_H_2_O]^−^, 203.0714 [M-H-CO_2_-C_2_H_2_O]^−^, 161.0595 [M-H-CO_2_-2C_2_H_2_O]^−^, 151.0397 [M-H-C_7_H_6_O_3_]^−^	Puerh tea
23	Epigallocatechin-3-*O*-gallate	C_22_H_18_O_11_	6.76	459.0918 (0.2)	457.0787 (2.2)	289.0365 [M+H-C_7_H_6_O_5_]^+^, 139.0388	305.0658 [M-H-C_7_H_4_O_4_]^−^, 169.0123 [C_7_H_5_O_5_]^−^, 125.0237 [M-H-C_7_H_4_O_4_-C_9_H_8_O_4_]^−^	Puerh tea
28	Gallocatechin-3-*O*-gallate	C_22_H_18_O_11_	7.00	459.0920 (0)	457.0777 (1.6)	289.2269[M+H-C_7_H_6_O_5_]^+^, 139.1220	305.0636 [M-H-C_7_H_4_O_4_]^−^, 169.0137 [C_7_H_5_O_5_]^−^, 125.0244 [M-H-C_7_H_4_O_4_-C_9_H_8_O_4_]^−^	Puerh tea
33	Epicatechin-3-*O*-gallate	C_22_H_18_O_10_	7.86	443.097 (−0.2)	441.0830 (1.2)	291.0484 [M+H-C_7_H_4_O_4_]^+^, 191.0347, 123.0459	289.0711 [M-H-C_7_H_4_O_4_]^−^, 169.0131 [C_7_H_5_O_5_]^−^, 125.0243 [M-H-C_7_H_4_O_4_-C_9_H_8_O_3_]^−^	Puerh tea
35	Catechin-3-*O*-gallate	C_22_H_18_O_10_	8.01	443.0972 (0.3)	441.0824 (0.7)	291.0884 [M+H-C_7_H_4_O_4_]^+^, 273.0787, 139.0393, 123.0455	289.0710 [M-H-C_7_H_4_O_4_]^−^, 245.0819 [M-H-C_7_H_4_O_4_-CO_2_]^−^, 169.0129 [C_7_H_5_O_5_]^−^, 125.0244 [M-H-C_7_H_4_O_4_-C_9_H_8_O_3_]^−^	Puerh tea
**Alkaloids**								
2	Synephrine ^a,c^ C_9_H_13_NO_2_		2.72	168.1014 (−4.7)	ND	150.0913 [M+H-H_2_O]^+^, 135.0681 [M+H-H_2_O-CH_3_]^+^, 107.0500 [M+H-	ND	GCP
						H_2_O-CH_3_-CO]^+^, 91.0561, 77.0409, 65.0417		
8	Theobromine/Theophylline	C_7_H_8_N_4_O_2_	5.21	181.0721 (0.4)	179.0585 (5.7)	124.0500 [M+H-C_2_H_3_NO] +,96.0567 [M+H-C_2_H_3_NO-CO]^+^	164.0332 [M-H-CH_3_]^−^, 122.0354 [M-H-C_2_H_3_NO]^−^	Puerh tea
11	8-Oxocaffeine	C_8_H_10_N_4_O_3_	5.36	211.0824 (−0.9)	209.0689 (2.9)	196.0589 [M+H-CH_3_]^+^, 154.0581 [M+H-C_2_H_3_NO]^+^,	194.0432 [M-H-CH_3_]^−^, 137.0218 [M-H-C_2_H_3_NO-CH_3_]^−^	Puerh tea
19	Caffeine ^a^	C_8_H_10_N_4_O_2_	6.32	195.0877 (0.2)	ND	138.0658 [M+H-C_2_H_3_NO]^+^, 123.0425 [M+H-C_2_H_3_NO-CH_3_]^+^, 110.0714 [M+H-C_2_H_3_NO-CO]^+^	ND	Puerh tea
53	Citrusin III	C_36_H_53_N_7_O_9_	11.84	728.3982 (0.6)	726.3872 (4.4)	700.4052 [M+H-CO]^+^, 587.3161, 474.2319	696.3815, 590.3348	GCP
60	Citrusin I	C_34_H_53_N_7_O_9_	13.74	704.3975 (−0.4)	ND	686.3833 [M+H-H_2_O]^+^	ND	GCP
	**Limonoids**							
75	Limonin ^c^	C_26_H_30_O_8_	16.65	471.2013 (0)	ND	425.1982 [M+H-CH_2_O_2_]^+^, 161.0631	ND	GCP
	**Flavonoids**							
10	Ampelopsin	C_15_H_12_O_8_	5.33	321.0605 (−0.1)	319.046 (0.3)	183.0294 [M+H-C_7_H_6_O_3_]^+^, 139.0374 [M+H-C_8_H_6_O_4_]^+^	137.0239 [M-H-C_8_H_6_O_4_]^−^	Puerh tea
13	Luteolin-6,8-di-*C*-glucoside ^c^	C_27_H_30_O_16_	5.73	611.1599 (−1)	609.1477 (2.6)	593.1499 [M+H-H_2_O]^+^, 575.1404 [M+H-2H_2_O]^+^, 557.1410 [M+H−3H_2_O]^+^, 473.1021 [M+H-C_4_H_8_O_4_-H_2_O]^+^, 353.0650 [M+H-2C_4_H_8_O_4_-H_2_O]^+^	489.1057 [M-H-C_4_H_8_O_4_]^−^, 399.0745 [M-H-C_4_H_8_O_4_-C_3_H_6_O_3_]^−^, 369.0630 [M-H-2C_4_H_8_O_4_]^−^	GCP
18	Vicenin-2 ^c^	C_27_H_30_O_15_	6.21	595.1659 (0.2)	593.1531 (3.3)	577.1542 [M+H-H_2_O]^+^, 559.1450 [M+H-2H_2_O]^+^ 541.1356 [[M+H-3H_2_O]^+^	503.1220 [M-H-C_3_H_6_O_3_]^−^, 473.1112 [M-H-C_4_H_8_O_4_]^−^, 383.0790 [M-H-C_4_H_8_O_4_-C_3_H_6_O_3_]^−^, 353.0681 [M-H-2C_4_H_8_O_4_]^−^	GCP
20	Chrysoeriol-6,8-di-*C*-glucoside	C_28_H_32_O_16_	6.41	625.1762 (−0.3)	623.1641 (3.8)	607.1664 [M+H-H_2_O]^+^, 589.1550 [M+H-2H_2_O]^+^, 571.1434 [M+H-3H_2_O]^+^, 487.1228 [M+H-C_4_H_8_O_4_-H_2_O]^+^, 367.0839 [M+H-2C_4_H_8_O_4_-H_2_O]^+^	503.1236 [M-H-C_4_H_8_O_4_]^−^, 413.0898 [M-H-C_4_H_8_O_4_-C_3_H_6_O_3_]^−^, 383.0794 [M-H-2C_4_H_8_O_4_]^−^	GCP
21	Narirutin ^c^	C_27_H_32_O_14_	6.43	581.1855 (−1.8)	579.1376 (−1.1)	273.0742 [M+H-Glc-Rha]^+^,	ND	GCP
27	Isoorientin ^c^	C_21_H_20_O_11_	6.95	449.1079 (0.2)	447.0937 (1)	431.0971 [M+H-H_2_O]^+^, 413.0877 [M+H-2H_2_O]^+^, 395.0755 [M+H-3H_2_O]^+^, 329.0657 [M+H-C_4_H_8_O_4_]^+^, 299.0556 [M+H-C_8_H_6_O_3_]^+^	357.0623 [M-H-C_3_H_6_O_3_]^−^, 327.0505 [M-H-C_4_H_8_O_4_]^−^	GCP
29	Orientin ^c^	C_21_H_20_O_11_	7.18	449.1075 (−0.7)	447.0938 (2.1)	431.0972 [M+H-H_2_O]^+^, 413.0860 [M+H-2H_2_O]^+^, 395.0802 [M+H-3H_2_O]^+^, 329.0644 [M+H-C_4_H_8_O_4_]^+^, 299.0561 [M+H-C_8_H_6_O_3_]^+^	357.0623 [M-H-C_3_H_6_O_3_]^−^, 327.0505 [M-H-C_4_H_8_O_4_]^−^	GCP
30	Rutin ^a,c^	C_27_H_30_O_16_	7.54	611.1607 (0)	609.1479 (2.9)	465.1023 [M+H-Rha]^+^, 303.0498 [M+H-Rha-Glc]^+^	301.0352 [M-H-Rha-Glc]^−^	GCP, Puerh tea
31	Lonicerin ^c^	C_27_H_30_O_15_	7.60	595.1656 (−0.3)	593.1534 (3)	449.1063 [M+H-Rha]^+^, 287.0548 [M+H-Glc-Rha]^+^	285.0413 [M-H-Glc-Rha]^−^	GCP, Puerh tea
32	Apigenin-8-*C*-glucoside	C_21_H_20_O_10_	7.74	433.1128 (−0.2)	431.0992 (1.8)	415.1031 [M+H-H_2_O]^+^, 397.0910 [M+H-2H_2_O]^+^, 379.0809 [M+H-3H_2_O]^+^, 313.0705 [M+H-C_4_H_8_O_4_]^+^, 283.0601 [M+H-C_8_H_6_O_3_]^+^	341.0674 [M-H-C_3_H_6_O_3_]^−^, 311.0567 [M-H-C_4_H_8_O_4_]^−^, 283.0617 [M-H-C_4_H_8_O_4_-CO]^−^	GCP
34	Quercetin-3-*O*-glucoside	C_21_H_20_O_12_	7.95	465.1031 (0.7)	463.0887 (1)	303.0497 [M+H-Glc]^+^	301.0356 [M-H-Glc]^−^, 271.0249, 151.0024 [M-H-Glc-C_8_H_6_O_3_]^−^	Puerh tea
36	Diosmetin-6-*C*-glucoside	C_22_H_22_O_11_	8.08	463.1237 (−0.2)	461.1093 (0.8)	445.1231 [M+H-H_2_O]^+^, 427.1036 [M+H-2H_2_O]^+^, 409.0924 [M+H-3H_2_O]^+^, 343.0801 [M+H-C_4_H_8_O_4_]^+^, 313.0700 [M+H-C_8_H_6_O_3_]^+^	371.0829 [M-H-C_3_H_6_O_3_]^−^, 341.0686 [M-H-C_4_H_8_O_4_]^−^, 298.0489 [M-H-C_4_H_8_O_4_-CO-CH_3_]^−^	GCP
37	Kaempferol-3-*O*-rutinoside	C_27_H_30_O_15_	8.15	595.1652 (−0.1)	593.1529 (2.9)	449.1063 [M+H-Rha]^+^, 287.0548 [M+H-Rha-Glc]^+^	285.0406 [M-H-Glc-Rha]^−^	Puerh tea
39	Naringin ^a,c^	C_27_H_32_O_14_	8.25	581.1868 (0.6)	597.1743 (4.1)	419.1312 [M+H-Glc]^+^, 273.0762 [M+H-Rha-Glc]^+^, 153.0179 [M+H-Rha-Glc-C_8_H_8_O]^+^	271.0615 [M-H-Glc-Rha]^−^, 151.0027 [M-H-Rha-Glc-C_8_H_8_O]^−^	GCP
40	Rhoifolin ^a,c^	C_27_H_30_O_14_	8.30	579.1713 (0.8)	577.158 (2.9)	433.1121 [M+H-Rha]^+^, 271.0599 [M+H-Rha-Glc]^+^	269.0455 [M-H-Rha-Glc]^−^	GCP
41	Diosmin ^c^	C_28_H_32_O_15_	8.47	609.1868 (−0.4)	607.1687 (3.4)		299.0566 [M-H-Glc-Rha]^−^, 284.0333 [M-H-Glc-Rha-CH_3_]^−^	GCP
42	Kaempferol-3-*O*-glucoside	C_21_H_20_O_11_	8.59	449.108 (0.4)	447.0944 (1)	287.0561 [M+H-Glc]^+^	284.0329 [M-H-Glc]^−^, 255.0300 [M-H-Glc-CHO]^−^, 227.0344 [M-H-Glc-CHO-CO]^−^,	Puerh tea
43	Neodiosmin	C_28_H_32_O_15_	8.60	609.1811 (−0.4)	607.1684 (2.6)	463.1253 [M+H-Rha]^+^, 301.0717 [M+H-Rha-Glc]^+^, 286.0465 [M+H-Rha-Glc-CH_3_]^+^	299.0569 [M-H-Glc-Rha]^−^, 284.0325 [M-H-Glc-Rha-CH_3_]^−^	GCP
44	Hesperidin ^a,c^	C_28_H_34_O_15_	8.64	611.1968 (−0.3)	609.1849 (3.7)	449.1425 [M+H-Glc]^+^, 303.0864 [M+H-Rha-Glc]^+^, 153.0181 [M+H-Rha-Glc-C_9_H_10_O_2_]^+^	301.0729 [M-H-Rha-Glc]^−^, 286.0496 [M-H-Rha-Glc-CH_3_]^−^	GCP
45	Homoeriodictyol ^c^	C_16_H_14_O_6_	8.68	303.0863 (0.1)	301.0722 (0.6)	153.0177 [M+H-C_9_H_10_O_2_]^+^, 117.0337 [M+H-C_9_H_10_O_2_-2H_2_O]^+^	286.0511 [M-H-CH_3_]^−^, 151.0032 [M-H-C_9_H_10_O_2_]^−^	GCP
47	5,3′-Dihydroxy-7,4′-dimethoxyflavone ^c^	C_17_H_14_O_6_	9.54	315.0856 (−2)	ND	300.0617 [M+H-CH_3_]^+^, 285.0427 [M+H-2CH_3_]^+^,	ND	GCP
48	Myricetin	C_15_H_10_O_8_	9.57	319.0447 (−0.4)	317.0312 (2.3)	273.0387 [M+H-H_2_O-CO]^+^, 245.0457 [M+H-H_2_O-2CO]^+^, 153.0185 [M+H-C_8_H_6_O_4_]^+^	271.0213 [M-H-H_2_O-CO]^−^, 178.9975 [M-H-C_7_H_6_O_3_]^−^, 151.0024 [M-H-C_8_H_6_O_4_]^−^	Puerh tea
49	Poncirin ^a^	C_28_H_34_O_14_	10.63	595.2021 (0)	593.1903 (3.8)	449.1415 [M+H-Rha]^+^, 287.0914 [M+H-Glc-Rha]^+^, 153.0170 [M+H-Glc-Rha-C_9_H_10_O]^+^	285.0768 [M-H-Glc-Rha]^−^	GCP
50	Isosakuranetin ^c^	C_16_H_14_O_5_	10.69	287.0913 (−0.5)	285.0775 (2.8)	153.0173 [M+H-C_9_H_10_O]^+^, 133.0633 [M+H-C_7_H_4_O_4_]^+^	243.0632 [M-H-C_2_H_3_O]^−^,	GCP
51	Luteolin	C_15_H_10_O_6_	11.40	287.0549 (−0.4)	285.0409 (1.1)	153.0203 [M+H-C_8_H_6_O_2_]^+^	133.0290 [M-H-C_7_H_4_O_4_]^−^	Puerh tea
52	Quercetin ^c^	C15H10O7	11.51	303.0501 (0.4)	301.0365 (3.8)	285.0372 [M+H-H_2_O]^+^, 257.0441 [M+H-H_2_O-CO]^+^, 229.0498 [M+H-H_2_O-2CO]^+^, 153.0177 [M+H-C_8_H_6_O_3_]^+^	178.9976 [M-H-C_7_H_6_O_2_]^−^, 151.0027 [M-H-C_8_H_6_O_3_]^−^	Puerh tea
54	Monohydroxy-trimethoxyflavone	C_18_H_16_O_6_	12.25	329.1019 (−0.1)	ND	314.0820 [M+H-CH_3_]^+^, 299.0540 [M+H-2CH_3_]^+^, 271,0580 [M+H-2CH_3_-CO]^+^, 181.0096, 153.0138	ND	GCP
55	7-Hydroxy-3,5,6,8-tetramethoxyflavone	C_19_H_18_O_7_	12.69	359.113 (1.3)	ND	344.0912 [M+H-CH_3_]^+^, 329.0663 [M+H-2CH_3_]^+^	ND	GCP
56	Naringenin ^a,c^	C_15_H_12_O_5_	13.16	273.0758 (0.1)	271.0626 (2.5)	153.0185 [M+H-C_8_H_8_O]^+^, 147.0442, 119.0500	151.0032 [M-H-C_8_H_8_O]^−^, 119.0505 [M-H-C_7_H_4_O_4_]^−^, 107.0143 [M-H-C_8_H_8_O-CO_2_]^−^	GCP
57	Apigenin ^a, c^	C_15_H_10_O_5_	13.30	271.0601 (0.1)	269.0466 (3.9)	153.0175 [M+H-C_8_H_6_O]^+^	151.003 [M-H-C_8_H_6_O]^+^	Puerh tea
58	7-Hydroxy-5,6,8,4′-tetramethoxyflavone ^c^	C_19_H_18_O_7_	13.65	359.1125 (−0.1)	ND	344.0851 [M+H-CH_3_]^+^, 329.0633 [M+H-2CH_3_]^+^, 326.0779 [M+H-CH_3_-H_2_O]^+^, 298.0814 [M+H-CH_3_-CO-H_2_O]^+^,	ND	GCP
59	Kaempferol ^a,c^	C_15_H_10_O_6_	13.67	287.055 (0.1)	285.0413 (4.5)	153.0170 [M+H-C_8_H_6_O_2_]^+^		Puerh tea
61	Hesperetin ^a,c^	C_16_H_14_O_6_	13.76	303.0865 (0.7)	301.0724 (3.4)	177.0546, 153.0177 [M+H-C_9_H_10_O_2_] +	286.0484 [M-H-CH_3_]^−^, 151.0022 [M-H-C_9_H_10_O_2_]^−^	GCP
62	Chrysoeriol ^c^	C_16_H_12_O_6_	13.76	301.0709 (0.9)	299.0571 (3.2)	286.0466 [M+H-CH_3_]^+^, 258.0526 [M+H-CH_3_-CO]^+^, 229.0485 [M+H-CO_2_-CO]^+^, 153.0152 [M+H-C_9_H_8_O_2_]^+^	284.0328 [M-H-CH_3_]^−^, 256.0393 [M-H-CH_3_-CO]^−^, 227.0352 [M-H-CO_2_-CO]^−^, 151.0010 [M-H-C_9_H_8_O_2_]^−^	GCP
63	5-Hydroxy-3,6,7,8-tetramethoxyflavone	C_19_H_18_O_7_	14.12	359.1126 (0.3)	ND	344.0904 [M+H-CH_3_]^+^, 329.0614 [M+H-2CH_3_]^+^ 298.0815 [M+H-CH_3_-CO-H_2_O]^+^	ND	GCP
64	5,6,7,3′,4′-Pentamethoxyflavanone	C_20_H_22_O_7_	14.36	375.1441 (0.8)	ND	211.0594 [M+H-C_10_H_10_O_2_]^+^, 196.0361 [M+H-C_10_H_10_O_2_-CH_3_]^+^, 150.0311 [M+H-C_10_H_10_O_2_-CO-CH_3_-H_2_O]^+^	ND	GCP
65	7-Hydroxy-5,6,8,3′,4′-pentamethoxyflavone	C_20_H_20_O_8_	14.52	389.1233 (0.4)	ND	374.0990 [M+H-CH_3_]^+^, 359.0759 [M+H-2CH_3_]^+^, 341.0635 [M+H-2CH_3_-H_2_O]^+^, 197.0073 [M+H-C_10_H_10_O_2_-2CH_3_]^+^	ND	GCP
66	3′-Hydroxy-5,6,7,8,4′-pentamethoxyflavone/4′-Hydroxy-5,6,7, 8,3′-pentamethoxyflavone	C_20_H_20_O_8_	14.53	389.1233 (0.4)	ND	374.0990 [M+H-CH_3_]^+^, 359.0759 [M+H-2CH_3_]^+^, 344.0635 [M+H-3CH_3_]^+^	ND	GCP
67	6-Hydroxy-5,7,8,4′-tetramethoxyflavone	C_19_H_18_O_7_	14.80	359.1124 (−0.3)	ND	344.0866 [M+H-CH_3_]^+^, 329.0637 [M+H-2CH_3_]^+^, 314.0393 [M+H-3CH_3_]^+^, 183.0314	ND	GCP
68	Isosinensetin ^c^ (3′,4′,5,7,8-pentamethoxyflavone)	C_20_H_20_O_7_	15.16	373.1285 (0.9)	ND	358.1048 [M+H-CH_3_]^+^, 343.0811 [M+H-2CH_3_]^+^, 315.0865 [M+H-2CH_3_-CO]^+^	ND	GCP
69	Monohydroxy-hexamethoxyflavone	C_21_H_22_O_9_	15.35	419.1335 (−0.4)	ND	404.1055 [M+H-CH_3_]^+^, 389.0878 [M+H-2CH_3_]^+^	ND	GCP
70	Monohydroxy-pentamethoxyflavanone	C_20_H_22_O_8_	15.49	391.1385 (−0.6)	ND	241.0709 [M+H-C_9_H_10_O_2_]^+^, 226.0452 [M+H-C_9_H_10_O_2_-CH_3_]^+^, 211.0249 [M+H-C_9_H_10_O_2_-2CH_3_]^+^, 183.0300 [M+H-C_9_H_10_O_2_-2CH_3_-CO]^+^	ND	GCP
71	5-Hydroxy-6,7,8,4′-tetramethoxyflavone	C_19_H_18_O_7_	15.51	359.1133 (2.2)	ND	329.0656 [M+H-2CH_3_]^+^	ND	GCP
72	5-Hydroxy-7,8,3′,4′-tetramethoxyflavone	C_19_H_18_O_7_	16.21	359.1129 (1)	ND	344.0908 [M+H-CH_3_]^+^, 329.0672 [M+H-2CH_3_]^+^, 311.0543 [M+H-2CH_3_-H_2_O]^+^, 197.0043 [M+H-C_10_H_10_O_2_]^+^,169.0114 [M+H-C_10_H_10_O_2_-CO]^+^	ND	GCP
73	5,7,3′,4′-Tetramethoxyflavone	C_19_H_18_O_6_	16.22	343.1178 (0.5)	ND	328.0944 [M+H-CH_3_]^+^, 327.0881 [M+H-CH_4_]^+^, 312.0610 [M+H-CH_4_-CH_3_]^+^, 299.0894 [M+H-CH_4_-CO]^+^, 283.0562 [M+H-2CH_4_-CO]^+^	ND	GCP
74	Sinensetin ^a,c^	C_20_H_20_O_7_	16.60	373.1287 (1.3)	ND	358.1072 [M+H-CH_3_]^+^, 357.0983 [M+H-CH_4_]^+^, 343.0829 [M+H-2CH_3_]^+^, 315.0868 [M+H-2CH_3_-CO]^+^	ND	GCP
76	5,6,7,4′-Tetramethoxyflavone	C_19_H_18_O_6_	16.83	343.1178 (0.5)	ND	328.0946 [M+H-CH_3_]^+^, 327.0846 [M+H-CH_4_]^+^, 313.0710 [M+H-2CH_3_]^+^, 299.0918 [M+H-CH_4_-CO]^+^, 285.0763 [M+H-2CH_3_-CO]^+^, 153.0185	ND	GCP
77	5,7,8,3′,4′-Pentamethoxyflavanone	C_20_H_22_O_7_	16.95	375.1438 (0)	ND	211.0594 [M+H-C_10_H_10_O_2_]^+^, 196.0362 [M+H-C_10_H_10_O_2_-CH_3_]^+^, 168.0406 [M+H-C_11_H_10_O_3_-CH_3_]^+^	ND	GCP
78	Dihydroxy-trimethoxyflavone	C_18_H_16_O_7_	17.57	345.0972 (1)	ND	330.0747 [M+H-CH_3_]^+^, 315.0490 [M+H-2CH_3_]^+^, 301.0706 [M+H-CO_2_]^+^	ND	GCP
79	5,6,7,8,3′,4′-Hexamethoxyflavanone	C_21_H_24_O_8_	17.76	405.1547 (0.8)	ND	241.0705 [M+H-C_10_H_10_O_2_]^+^, 226.0464 [M+H-C_10_H_10_O_2_-CH_3_]^+^, 211.0233 [M+H-C_10_H_10_O_2_-2CH_3_]^+^, 183.0287	ND	GCP
80	5,7,4′-Trimethoxyflavone	C_18_H_16_O_5_	18.07	313.1084 (4.4)	ND	298.0896 [M+H-CH_3_]^+^, 270.0929 [M+H-CO-CH_3_]^+^, 269.0823 [M+H-CO_2_]^+^	ND	GCP
81	Nobiletin ^a,c^	C_21_H_22_O_8_	18.09	403.1391 (0.8)	ND	388.1145 [M+H-CH_3_]^+^, 373.0905 [M+H-2CH_3_]^+^, 358.0677 [M+H-3CH_3_]^+^, 327.0853 [M+H-3CH_3_-OCH_3_]^+^	ND	GCP
82	Dihydroxy-tetramethoxyflavone	C_19_H_18_O_8_	18.16	375.1072 (0.9)	ND	360.0817 [M+H-CH_3_]^+^, 345.0568 [M+H-2CH_3_]^+^, 330.0371 [M+H-3CH_3_]^+^, 327.0484 [M+H-2CH_3_-H_2_O]^+^, 197.0088	ND	GCP
83	5,7,8,4′-Tetramethoxyflavone	C_19_H_18_O_6_	18.39	343.118 (1.2)	ND	327.0862 [M+H-CH_4_]^+^, 313.0710 [M+H-2CH_3_]^+^, 285.0751 [M+H-2CH_3_-CO]^+^, 282.0890 [M+H-2CH_3_-OCH_3_]^+^, 153.0179	ND	GCP
84	Monohydroxy-tetramethoxyflavanone	C_19_H_20_O_7_	18.88	361.1284 (0.5)	ND	197.0425 [M+H-C_10_H_12_O_2_]^+^, 182.0205 [M+H-C_10_H_12_O_2_-CH_3_]^+^, 136.0151	ND	GCP
85	3,5,6,7,8,3′,4′-Heptemethoxyflavone	C_22_H_24_O_9_	19.08	433.1496 (0.8)	ND	418.1268 [M+H-CH_3_]^+^, 403.1024 [M+H-2CH_3_]^+^, 385.0925 [M+H-2CH_3_-H_2_O]^+^, 345.0610 [M+H-4CH_3_-CO]^+^	ND	GCP
86	5-Hydroxy-6,7,8,3′,4′-pentamethoxyflavanone	C_20_H_22_O_8_	19.56	391.139 (0.7)	ND	227.0535 [M+H-C_10_H_12_O_2_]^+^, 212.0306 [M+H-C_10_H_12_O_2_-CH_3_] +,149.0224	ND	GCP
87	Monohydroxy-tetramethoxyflavone	C_19_H_18_O_7_	19.65	359.1124 (−0.3)	ND	344.0900 [M+H-CH_3_]^+^, 326.0762 [M+H-CH_3_-H_2_O]^+^, 298.0828 [M+H-CH_3_-CO-H_2_O]^+^, 162.0676	ND	GCP
88	Tangeretin ^a,c^	C_20_H_20_O_7_	19.88	373.1286 (1.1)	ND	358.1049 [M+H-CH_3_]^+^, 343.0810 [M+H-2CH_3_]^+^, 328.0584 [M+H-3CH_3_]^+^, 325.0715 [M+H-2CH_3_-H_2_O]^+^, 315.0868 [M+H-2CH_3_-CO]^+^	ND	GCP
89	Monohydroxy-tetramethoxyflavone	C_19_H_18_O_7_	20.50	359.1125 (0)	ND	344.0867 [M+H-CH_3_]^+^, 343.0808 [M+H-CH_4_]^+^, 315.0845 [M+H-CO_2_]^+^, 164.0841	ND	GCP
90	5-Hydroxy-6,7,8,3′,4′-pentamethoxyflavone	C_20_H_20_O_8_	21.20	389.1234 (0.9)	ND	374.0984 [M+H-CH_3_]^+^, 359.0756 [M+H-2CH_3_]^+^, 341.0658 [M+H-2CH_3_-H_2_O]^+^, 197.0088 [M+H-C_10_H_12_O_2_-2CH_3_]^+^	ND	GCP
91	Natsudaidain	C_21_H_22_O_9_	22.26	419.1337 (0.1)	ND	404.1168 [M+H-CH_3_]^+^, 389.0870 [M+H-2CH_3_]^+^, 371.0800 [M+H-2CH_3_-H_2_O]^+^	ND	GCP
92	Monohydroxy-tetramethoxyflavone	C_19_H_18_O_7_	23.02	359.1127 (−0.3)	ND	344.0886 [M+H-CH_3_]^+^, 329.0654 [M+H-2CH_3_]^+^, 311.0552 [M+H-2CH_3_-H_2_O]^+^, 197.0069	ND	GCP

^a^ Confirmation in comparison with authentic standards. ^b^ The losses are: Glc = glucose moiety, Rha = rhamnose moiety. ND = not detect. ^c^ Confirmation in comparison with mass spectral library (Natural Products HR-MS/MS Spectral Library, Version 1.0; AB Sciex, Foster City, CA, USA). ^d^ ND = not detect.

## References

[1] Lv H.-P., Zhang Y.-J., Lin Z., Liang Y.-R. (2013). Processing and chemical constituents of Pu-erh tea: A review. Food Res. Int..

[2] Duh P.D., Yen G.C., Yen W.J., Wang B.S., Chang L.W. (2004). Effects of pu-erh tea on oxidative damage and nitric oxide scavenging. J. Agric. Food Chem..

[3] Kuo K.L., Weng M.S., Chiang C.T., Tsai Y.J., Lin-Shiau S.Y., Lin J.K. (2005). Comparative studies on the hypolipidemic and growth suppressive effects of oolong, black, pu-erh, and green tea leaves in rats. J. Agric. Food Chem..

[4] Zheng Y., Zeng X., Peng W., Wu Z., Su W. (2018). Study on the discrimination between Citri Reticulatae Pericarpium varieties based on HS-SPME-GC-MS combined with multivariate statistical analyses. Molecules.

[5] Yu X., Sun S., Guo Y., Liu Y., Yang D., Li G., Lu S. (2018). Citri Reticulatae Pericarpium (Chenpi): Botany, ethnopharmacology, phytochemistry, and pharmacology of a frequently used traditional Chinese medicine. J. Ethnopharmacol..

[6] Zheng Y., Zeng X., Peng W., Wu Z., Su W. (2019). Characterisation and classification of Citri Reticulatae Pericarpium varieties based on UHPLC-Q-TOF-MS/MS combined with multivariate statistical analyses. Phytochem. Anal..

[7] Fu M., Xu Y., Chen Y., Wu J., Yu Y., Zou B., An K., Xiao G. (2017). Evaluation of bioactive flavonoids and antioxidant activity in Pericarpium Citri Reticulatae (Citrus reticulata ‘Chachi’) during storage. Food Chem..

[8] Luo Y., Zeng W., Huang K.E., Li D.X., Chen W., Yu X.Q., Ke X.H. (2019). Discrimination of Citrus reticulata Blanco and Citrus reticulata ‘Chachi’ as well as the Citrus reticulata ‘Chachi’ within different storage years using ultra high performance liquid chromatography quadrupole/time-of-flight mass spectrometry based metabolomics approach. J. Pharm. Biomed. Anal..

[9] Valdes A.M., Walter J., Segal E., Spector T.D. (2018). Role of the gut microbiota in nutrition and health. BMJ.

[10] Feng Q., Chen W.D., Wang Y.D. (2018). Gut microbiota: An integral moderator in health and disease. Front. Microbiol..

[11] Turnbaugh P.J., Hamady M., Yatsunenko T., Cantarel B.L., Duncan A., Ley R.E., Sogin M.L., Jones W.J., Roe B.A., Affourtit J.P. (2009). A core gut microbiome in obese and lean twins. Nature.

[12] Lambeth S.M., Carson T., Lowe J., Ramaraj T., Leff J.W., Luo L., Bell C.J., Shah V.O. (2015). Composition, diversity and abundance of gut microbiome in prediabetes and type 2 diabetes. J. Diabetes Obes..

[13] Guinane C.M., Cotter P.D. (2013). Role of the gut microbiota in health and chronic gastrointestinal disease: Understanding a hidden metabolic organ. Ther. Adv. Gastroenterol..

[14] Feng W., Ao H., Peng C., Yan D. (2019). Gut microbiota, a new frontier to understand traditional Chinese medicines. Pharmacol. Res..

[15] Manach C., Scalbert A., Morand C., Remesy C., Jimenez L. (2004). Polyphenols: Food sources and bioavailability. Am. J. Clin. Nutr..

[16] Tanja M., Salzberg S.L. (2011). FLASH: Fast length adjustment of short reads to improve genome assemblies. Bioinformatics.

[17] Bolger A.M., Marc L., Bjoern U. (2014). Trimmomatic: A flexible trimmer for Illumina sequence data. Bioinformatics.

[18] Edgar R.C. (2010). Search and clustering orders of magnitude faster than BLAST. Bioinformatics.

[19] Caporaso J.G., Kuczynski J., Stombaugh J., Bittinger K., Bushman F.D., Costello E.K., Fierer N., Peña A.G., Goodrich J.K., Gordon J.I. (2010). QIIME allows analysis of high-throughput community sequencing data. Nat. Methods.

[20] Han T.X., Xu X.Y., Zhang M.J., Peng X., Du L.L. (2010). Global fitness profiling of fission yeast deletion strains by barcode sequencing. Genome Biol..

[21] Zeng X., Su W., Bai Y., Chen T., Yan Z., Wang J., Su M., Zheng Y., Peng W., Yao H. (2017). Urinary metabolite profiling of flavonoids in Chinese volunteers after consumption of orange juice by UFLC-Q-TOF-MS/MS. J. Chromatogr. B.

[22] Zeng X., Su W., Zheng Y., Liu H., Li P., Zhang W., Liang Y., Bai Y., Peng W., Yao H. (2018). UFLC-Q-TOF-MS/MS-based screening and identification of flavonoids and derived metabolites in human urine after oral administration of Exocarpium Citri Grandis extract. Molecules.

[23] Liu Z., Chen Z., Guo H., He D., Zhao H., Wang Z., Zhang W., Liao L., Zhang C., Ni L. (2016). The modulatory effect of infusions of green tea, oolong tea, and black tea on gut microbiota in high-fat-induced obese mice. Food Funct..

[24] Sharma M., Akhtar N., Sambhav K., Shete G., Bansal A.K., Sharma S.S. (2015). Emerging potential of citrus flavanones as an antioxidant in diabetes and its complications. Curr. Top. Med. Chem..

[25] Scalbert A., Manach C., Morand C., Remesy C., Jimenez L. (2005). Dietary polyphenols and the prevention of diseases. Crit. Rev. Food Sci. Nutr..

[26] Sun Y., Wang J., Gu S., Liu Z., Zhang Y., Zhang X. (2010). Simultaneous determination of flavonoids in different parts of Citrus reticulata ‘Chachi’ fruit by high performance liquid chromatography-photodiode array detection. Molecules.

[27] Zhao M., Xiao W., Ma Y., Sun T., Yuan W., Tang N., Zhang D., Wang Y., Li Y., Zhou H. (2013). Structure and dynamics of the bacterial communities in fermentation of the traditional Chinese post-fermented pu-erh tea revealed by 16S rRNA gene clone library. World J. Microbiol. Biotechnol..

[28] Zhang G., Zeng G., Cai X., Deng S., Luo H., Sun G. (2007). Brachybacterium zhongshanense sp. nov., a cellulose-decomposing bacterium from sediment along the Qijiang River, Zhongshan City, China. Int. J. Syst. Evol. Microbiol..

[29] Williams B.A., Grant L.J., Gidley M.J., Mikkelsen D. (2017). Gut fermentation of dietary fibres: Physico-chemistry of plant cell walls and implications for health. Int. J. Mol. Sci..

[30] Kemperman R.A., Gross G., Mondot S., Possemiers S., Marzorati M., Van de Wiele T., Doré J., Vaughan E.E. (2013). Impact of polyphenols from black tea and red wine/grape juice on a gut model microbiome. Food Res. Int..

[31] Mosele J.I., Gosalbes M.J., Macia A., Rubio L., Vazquez-Castellanos J.F., Jimenez Hernandez N., Moya A., Latorre A., Motilva M.J. (2015). Effect of daily intake of pomegranate juice on fecal microbiota and feces metabolites from healthy volunteers. Mol. Nutr. Food Res..

[32] Etxeberria U., Hijona E., Aguirre L., Milagro F.I., Bujanda L., Rimando A.M., Martinez J.A., Portillo M.P. (2017). Pterostilbene-induced changes in gut microbiota composition in relation to obesity. Mol. Nutr. Food Res..

[33] Koh A., De Vadder F., Kovatcheva-Datchary P., Backhed F. (2016). From dietary fiber to host physiology: Short-chain fatty acids as key bacterial metabolites. Cell.

[34] Chen C.-C., Walker W.A. (2005). Probiotics and prebiotics: Role in clinical disease states. Adv. Pediatr..

[35] Shang Q., Shan X., Cai C., Hao J., Li G., Yu G. (2016). Dietary fucoidan modulates the gut microbiota in mice by increasing the abundance of Lactobacillus and Ruminococcaceae. Food Funct..

[36] Pozuelo M., Panda S., Santiago A., Mendez S., Accarino A., Santos J., Guarner F., Azpiroz F., Manichanh C. (2015). Reduction of butyrate- and methane-producing microorganisms in patients with Irritable Bowel Syndrome. Sci. Rep..

[37] Menni C., Lin C.H., Cecelja M., Mangino M., Matey-Hernandez M.L., Keehn L., Mohney R.P., Steves C.J., Spector T.D., Kuo C.F. (2018). Gut microbial diversity is associated with lower arterial stiffness in women. Eur. Heart J..

[38] Kang C., Wang B., Kaliannan K., Wang X., Lang H., Hui S., Huang L., Zhang Y., Zhou M., Chen M. (2017). Gut microbiota mediates the protective effects of dietary capsaicin against chronic low-grade inflammation and associated obesity induced by high-fat diet. mBio.

[39] Mancabelli L., Milani C., Lugli G.A., Turroni F., Mangifesta M., Viappiani A., Ticinesi A., Nouvenne A., Meschi T., van Sinderen D. (2017). Unveiling the gut microbiota composition and functionality associated with constipation through metagenomic analyses. Sci. Rep..

[40] Li L., Batt S.M., Wannemuehler M., Dispirito A., Beitz D.C. (1998). Effect of feeding of a cholesterol-reducing bacterium, Eubacterium coprostanoligenes, to germ-free mice. Lab. Anim. Sci..

[41] Candela M., Perna F., Carnevali P., Vitali B., Ciati R., Gionchetti P., Rizzello F., Campieri M., Brigidi P. (2008). Interaction of probiotic Lactobacillus and Bifidobacterium strains with human intestinal epithelial cells: Adhesion properties, competition against enteropathogens and modulation of IL-8 production. Int. J. Food Microbiol..

[42] Resta-Lenert S., Barrett K.E. (2003). Live probiotics protect intestinal epithelial cells from the effects of infection with enteroinvasive Escherichia coli (EIEC). Gut.

[43] Masood M.I., Qadir M.I., Shirazi J.H., Khan I.U. (2011). Beneficial effects of lactic acid bacteria on human beings. Crit. Rev. Microbiol..

[44] Llopis M., Antolin M., Carol M., Borruel N., Casellas F., Martinez C., Espin-Basany E., Guarner F., Malagelada J.R. (2009). Lactobacillus casei downregulates commensals’ inflammatory signals in Crohn’s disease mucosa. Inflamm. Bowel Dis..

[45] Picard C., Fioramonti J., Francois A., Robinson T., Neant F., Matuchansky C. (2005). Review article: Bifidobacteria as probiotic agents—Physiological effects and clinical benefits. Aliment. Pharmacol. Ther..

[46] Liu M., Zhang X., Hao Y., Ding J., Shen J., Xue Z., Qi W., Li Z., Song Y., Zhang T. (2019). Protective effects of a novel probiotic strain, Lactococcus lactis ML2018, in colitis: In vivo and in vitro evidence. Food Funct..

[47] LeBlanc J.G., Chain F., Martin R., Bermudez-Humaran L.G., Courau S., Langella P. (2017). Beneficial effects on host energy metabolism of short-chain fatty acids and vitamins produced by commensal and probiotic bacteria. Microb. Cell Fact..

[48] Yao J. (2013). Isolation and molecular identification of the bacterial colonization during the pile fermentation process of Pu-erh tea. J. Anhui Agric. Sci..

